# Hematopoietic stem cell transplantation in Zagreb: the first 40 years

**DOI:** 10.3325/cmj.2023.64.123

**Published:** 2023-04

**Authors:** Boris Labar

**Affiliations:** Professor Emeritus, University of Zagreb School of Medicine, Zagreb, Croatia

## Abstract

Hematopoietic stem cell transplantation is the most effective treatment for acute leukemia, severe aplastic anemia, and some hereditary hematological disorders. The principal source of stem cells in this procedure are bone marrow and peripheral blood cells. In recent years, the transplantation outcome has significantly improved. The availability of the donor no longer poses a problem, as transplantation has been performed routinely from related, unrelated, and haploidentical donors. A high success rate has been reported in elderly patients transplanted with reduced-intensity conditioning. Improved patient care has decreased toxicity and mortality after treatment. This article gives an overview of the 40-year history of the Zagreb transplant program. It also discusses the use of hematopoietic stem cell transplantation in various hematological disorders, with a special emphasis on the publications by the Zagreb transplant team.

Labar: Hematopoietic stem cell transplantation in Zagreb – the first 40 years

Correspondence to: Boris Labar Hegedušićeva 6 10 000 Zagreb, Croatia *boris.labar@inet.hr*

This year marks the 40th anniversary of the first hematopoietic stem cell transplantation (HSCT) in Zagreb (Figure 1). Over the last 40 years, the rates of bone marrow transplantation have grown exponentially. In the 1980s, fewer than 50 centers in the world were routinely performing HSCT. Today, more than 700 transplant teams from more than 70 countries all over the world send their data to the Registry of the European Blood and Marrow Transplant (EBMT) group. The availability of transplantation has also significantly improved. The use of related or unrelated human leukocyte antigen (HLA)-identical donors and haploidentical donors eliminated the problem of finding a suitable donor. The toxicity of treatment has significantly decreased compared with the 1980s. Diagnostics and treatment are more sensitive and prompt, especially in the case of infections, and transfusion support has significantly improved. The morbidity and mortality from graft-vs host disease (GVHD) have decreased. So, today HSCT is a routine treatment approach for many hematological diseases.

**Figure 1:**
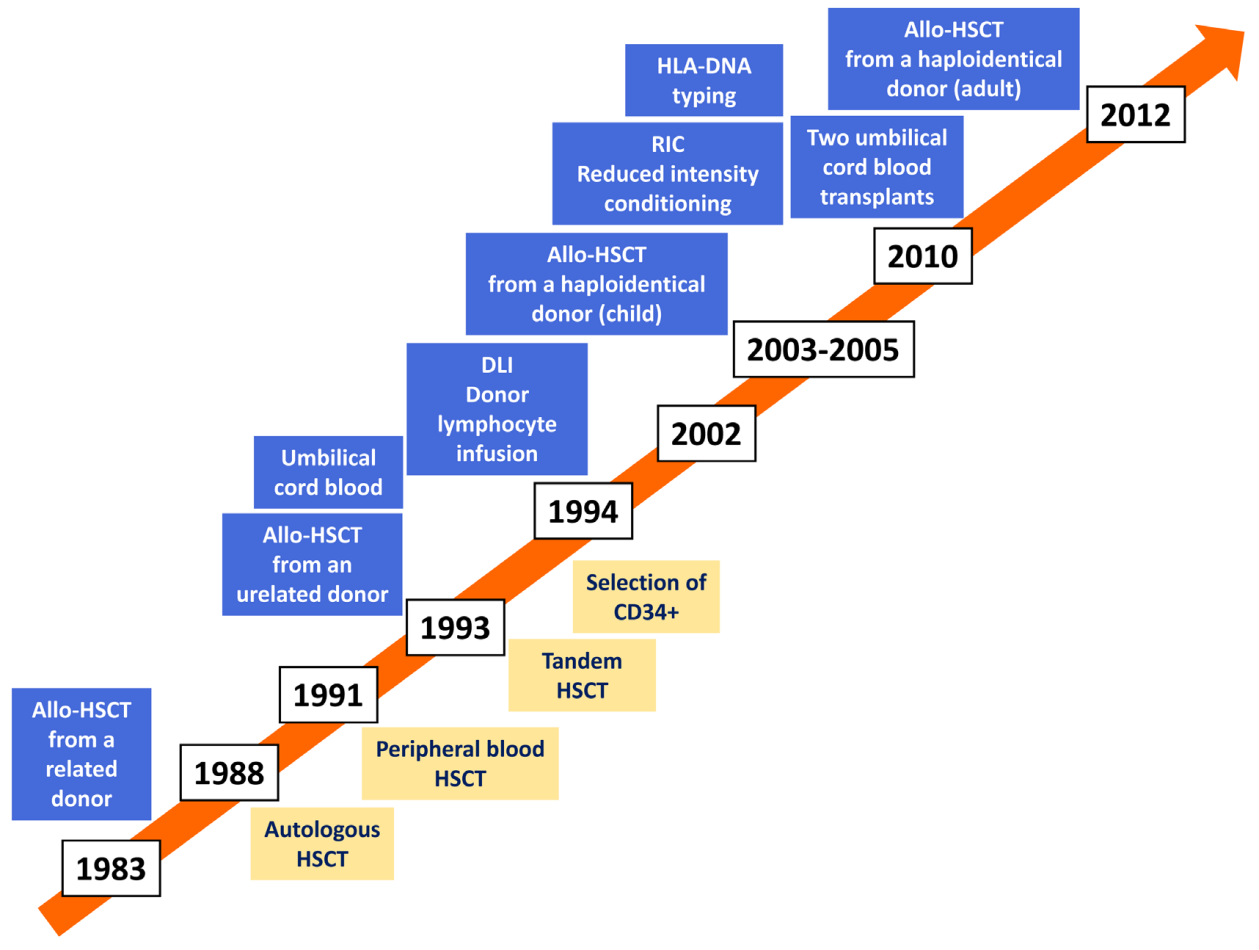
The timeline of important developments related to hematopoietic stem cell transplantation (HCST) in Croatia.

Transplantation is the treatment of choice for some hereditary hematologic disorders and for young patients with severe aplastic anemia. It is also the most effective antileukemia treatment. In recent years, our knowledge of the pathogenesis of hematological neoplasms has significantly improved, allowing more appropriate prognostic characterization of hematological neoplasms and better patient treatment.

## The beginning of the transplant program in Zagreb

The HSCT program in Zagreb started in February 1983. At that time, transplantation experienced a transition from an experimental to a clinical field. The first studies on mice in 1957 showed that leukemia could be eliminated after total body irradiation and infusion of homologous (allogeneic) or heterologous (xenogeneic) bone marrow and lymphoid cells but not of isologous (syngeneic) cells (1). Irradiation alone did not eliminate the disease. It was speculated that homologous bone marrow from a different strain of mouse might be able to destroy, by immune reaction, residual leukemic cells as well as those of the host. This was the beginning of research on graft-vs-leukemia/graft-vs-host (GVL/GVH) reaction. GVH reaction occurs when the donor’s immune cells initiate a reaction that damages host organs and tissues. At the same time, they destroy the residual leukemic cells (GVL).

One year later, in 1958, following an accident at the Vinča Nuclear Reactor, Yugoslavia, six workers were exposed to very high doses of radiation. One died shortly afterwards and the other five were allografted in Paris by the French hematologist G. Mathe (2). They received bone marrow from French volunteers and survived. But at that time, nobody knew that a successful engraftment required an HLA match. So, there is still a doubt whether the transplanted bone marrow was the actual reason why the victims survived.

In leukemia treatment, the first trials on bone marrow transplantation were disappointing. Thomas (3) treated a group of patients who had end-stage leukemia with high-dose radiation and a bone marrow transplant. All patients died within a hundred days. However, in two patients it was proven for the first time that cells circulating in the blood following transplantation were of the donor type. Thomas did not give up. In the next few years, two crucial moments occurred that changed the future of HSCT. The first was the discovery of HLA (4). The second was Thomas’s study on HSCT in patients who had an early phase of the disease. In these patients, the long-term disease-free survival approached 50% (5). These developments paved the way for a new era of clinical HSCT in the mid-70s. In less than 10 years, the Zagreb program was initiated.

The launching of the Zagreb program was facilitated by the scientific work of the Zagreb experimental bone marrow transplant team at the Ruđer Bošković Institute. Their research on HSCT in mice, especially on GVL/GVH reaction, greatly enhanced our understanding of HSCT. Silobrčić et al (6) and Vitale et al (7) defined the temporal phases of GVH reactivity: (i) homing and recognition, (ii) activation and proliferation, and (iii) emigration into and injury of target tissues. Boranić showed that a potentially lethal donor graft did not need to be permanently present. It could be replaced with a less aggressive one after leukemia cells had perished, a procedure he called “rescuing the host” (8-10).

In 1978, Boranić organized an advisory conference on HSCT in Croatia (11,12), at which it was decided that the minimal clinical conditions existed to start a HSCT program, and that the HSCT Center should be located at the University Hospital Center Zagreb.

Resources for the Center were partly covered by the Krško Nuclear Plant, which was under construction at the time, since bone marrow transplantation was planned as part of the tertiary medical care for the plant.

HSCT was not a priority in Croatian medicine at the time. Many physicians doubted the program, arguing that therapy for a few patients was not as important as, for example, vaccination for all. The arguments in favor of HSCT – the existence of another transplant program (kidney transplantation), very low cost of HSCT in Croatia compared with the prices abroad, and the fact that transplantation will hasten the development of clinical hematology (13) – did not convince the majority to accept HSCT. Despite the opposing views, many hematologists and clinicians recognized the value of such a program and gave us their support.

## HSCT for aplastic anemia

HSCT is the treatment of choice for patients with severe aplastic anemia (SAA) younger than 45 years with an HLA-identical sibling donor (14). More than 80% of these patients can be cured. The first-line treatment for older patients and for patients without a suitable donor is immunosuppressive therapy (IST) (15).

The first three patients in the Zagreb Center were allografted because of SAA (16). For immunosuppression, we used conditioning with a combination of cyclophosphamide and thoraco-abdominal irradiation, with shielding of the ovaries or testes. This enabled the engraftment and prevented the rejection of the donor marrow cells. Our first published results showed the cure rate of about 50% (16-18). The reason for the lower treatment outcome were unfavorable prognostic factors. Most of the patients received more than 50 transfusions before transplantation. They were also allografted very late in the course of the disease after several lines of therapies. These factors significantly influenced HSCT efficiency (19). Furthermore, immunosuppressive conditioning with thoraco-abdominal radiation was associated with late complications in terms of increased secondary neoplasms and myelodysplastic syndrome (MDS) (20). This is why we changed the conditioning to use a combination of cyclophosphamide and anti-thymocyte globulin (ATG). While IST and HSCT have a similar efficacy, reaching 70% or more (21), our results with IST were inferior compared with HSCT (22). The probability of long-term survival for patients receiving IST and HSCT was 40% and 60%, respectively. The IST group patients were reported to develop secondary neoplasms. Studies that applied molecular sequencing reported a large proportion of patients with clonal hematopoiesis and acquired mutations in myeloid leukemia genes (23,24). These mutations appeared early after IST and have increased in frequency over time. Some of these acquired mutations confer a very high risk of progression to MDS/acute myeloid leukemia (AML) (23,24).

The best source of stem cells for SAA patients undergoing HSCT is the bone marrow. It showed a superior outcome in patients receiving a transplant from an HLA-identical sibling compared with peripheral blood stem cells (25). The superior outcome could be attributed to a decreased incidence of chronic graft-vs-host disease (GVHD) and associated mortality (26). The possibility to find an HLA-identical sibling donor is limited. In the past, many investigators tried to expand the donor pool by investigating the efficacy of HSCT with an alternative donor. In the beginning, the treatment with an HLA-matched unrelated donor was disappointing (27). HSCT was associated with an inappropriately high morbidity and mortality because of graft rejection, GVHD, and interstitial pneumonitis. In recent years, the use of high-resolution 10/10-HLA-allele level matching donors and intensified conditioning (adding 2 Gy total body irradiation [TBI] or/and fludarabine to ATG and cyclophosphamide) has yielded comparable results to HLA-identical HSCT (28), especially in young patients and children (29).

In the past year, trials using another pool of donors, haploidentical donors, yielded very encouraging preliminary data. In this setting, it is appropriate to use post-transplantation cyclophosphamide and granulocyte-stimulating factor/ATG regimen. Two-year overall survival was 90% or more (30).

## HSCT for leukemia and lymphoma

Allogeneic HSCT is the most effective antileukemia treatment, able to change the disease outcome. Myeloablative conditioning regimen combined with GVL reaction leads to favorable outcomes in patients with leukemia – a significant proportion have a chance to be cured or to become long-term disease-free survivors. There are numerous reports about HSCT efficiency in AML (31-33). One of the first prospective studies in AML was conducted by the Leukemia Group of the European Organization for Research and Treatment of Cancer (EORTC) (34). They compared three postremission treatment regimens: allogeneic HSCT vs autologous HSCT vs chemotherapy. The result showed that transplantation better controlled AML than chemotherapy. The projected rate of disease-free survival (DFS) at four years was 55% for allogeneic HSCT, 48% for autologous HSCT, and 30% for intensive chemotherapy. The overall survival (OS) after complete remission (CR) was similar in all three groups, since more patients who relapsed in the chemotherapy group had a response to subsequent transplantation. According to intention-to-treat analysis, young allografted patients with unfavorable cytogenetics had a better DFS and OS compared with patients receiving autologous marrow (35).

Similar clinical studies have been conducted by the Zagreb transplant team. We also compared allogeneic transplantation and chemotherapy in patients with *de-novo* AML who achieved a first CR (36). Patients treated with allogeneic transplant had a significantly higher adjusted probability of leukemia-free survival (LFS) (68 ± 14 vs 24 ± 20 at 4 years). The better outcome in the HSCT group was related to a lower relapse rate (70% vs 20% at 4 years). In another study (37), patients who underwent allogeneic HSCT had higher LFS (68% vs 55%; *P* = ns) and a significantly lower relapse rate at three years (12% vs 38%, *P* = 0.02) , compared with the autologous group. Contrary to this, the allotransplant group had a higher transplant-related mortality (23% vs 14% at 5 years *P* = ns). The principal cause of death in the allogeneic HSCT group was GVHD with or without infection. The increased antileukemic effect in allotransplant patients was diminished by the toxicity of the transplantation procedure.

Allogeneic HSCT is also an effective treatment option to prevent relapse in adults with acute lymphoblastic leukemia (ALL). The use of allogeneic HSCT in the first CR is recommended for all patients with a high risk of relapse (38-40). The strongest predictor of relapse is the persistence of minimal residual disease (MRD) (41,42). It is mandatory to monitor MRD by using either molecular methods or flow cytometry. In patients with positive MRD, BMT is the treatment of choice. The preferred donors for allogeneic BMT are an HLA-identical sibling or a matched unrelated donor. Patients with a high risk of relapse lacking an HLA-matched donor could be offered transplantation from a mismatched related (including haploidentical) donor or an unrelated donor with a single HLA allele/antigen disparity. Patients with persistent MRD can achieve a very high rate of molecular remissions if treated with monoclonal antibodies, such as bispecific T-cell engager blinatumomab (43). However, it remains unclear whether this intervention may obviate allogeneic HSCT. Novel agents such as inotuzumab ozogamicin (a conjugate of anti-CD22 monoclonal antibody with calicheamicin) used as front-line therapy in combination with reduced-dose chemotherapy also increase the treatment efficacy (44). The need for allogeneic HSCT may be decreased by using the monoclonal antibody blinatumomab as consolidation (45). The studies with monoclonal antibodies in ALL are ongoing. Preliminary data with chimeric antigen receptor T cells (CAR-T) or natural killer cells are very encouraging (46,47). A combination of the mentioned options may in the future replace allogeneic HSCT.

The EORTC Leukemia group conducted a prospective study among patients ≤50 years old with ALL in the first CR (48). The efficacy of allogeneic HSCT was compared with that of autologous transplantation and/or maintenance chemotherapy. The six-year DFS was similar (38.2% vs 36.8% respectively). As in AML, the donor group had a lower relapse incidence but a higher cumulative incidence of death from toxicity.

An individual patient data meta-analysis by Gupta et al (49), including data from 81 institutions, showed that allogeneic, but not autologous HSCT, improved survival only among younger adults with ALL in the first remission.

Autologous transplantation is another option for the treatment of acute leukemia. Patients with standard or favorable prognosis treated in the first remission had a 40% chance for long-term survival (50-53). The Zagreb Center has been using autologous HSCT since 1988. Preliminary data (54,55) showed good antileukemia efficacy with low transplant-related toxicity. The problem was a high relapse rate. Now, autologous HSCT is a routine and effective treatment for lymphoma and multiple myeloma (56,57). In acute leukemia and myeloid neoplasms, autologous HSCT is not performed regularly. Allogeneic HSCT is the preferable therapy.

Recent results with haploidentical transplant in acute leukemia are very encouraging, approaching those with related and unrelated matched donors (58-61).

In older patients, the outcome of HSCT is still jeopardized by transplant-related mortality (TRM). In order to decrease TRM, reduced-intensity preparative regimens were used. These regimens led to full donor chimerism and generation of GVL effect with curative potential (62-64).

In the last decade, the discovery of genetic abnormalities has changed the classification and treatment approach to both AML and ALL. The prognostic factors for AML (65) are divided into patient-related factors (age and performance status), AML-related genetic factors, and factors after diagnosis. Adverse genetic factors and the persistence of MRD after initial treatment are indications for prompt allogeneic BMT. High-risk predictors of relapse in ALL (66) are older age, high WBC count, poor cytogenetic and molecular abnormalities, poor immunophenotyping markers, and failure to achieve CR in four weeks. These groups of patients should be allografted in the first CR.

For many years, allotransplant had been also the treatment of choice for chronic myeloid leukemia (CML) (67). After the introduction of tyrosine kinase inhibitors (TKI), allogeneic HSCT has become recommended as a second- or third-line therapy for patients in the first chronic phase or as salvage for patients with very advanced disease (68).

We reported the first successful umbilical cord blood (UCB) transplant in a child with CML, who received UCB from her sister (69). The patient relapsed after three years. She was then treated with donor buffy coat cells (donor lymphocytes to induce the GVL reaction) and interferon-α. After six months of treatment, complete hematologic, cytogenetic, and molecular remission was achieved (70).

UCB contains enough hematopoietic stem cells to reconstitute the hematopoietic compartment. It is collected at birth without causing harm to the newborn and could be cryopreserved and kept for a long time. UCB, together with bone marrow and peripheral blood, has become a valuable source of hematopoietic stem cells. Over 600 000 UCB units have been stored for transplantation worldwide, and more than 30 000 UCB transplantations have been performed. Zagreb also has its own UCB bank (71).

## HSCT is more than transplantation

In Zagreb, HSCT introduced a new concept of clinical work – team approach. The transplant team consists of experts from many different clinical disciplines. Team work was the only way to transfer new diagnostic and treatment modalities related to transplantation. The transplantation program in Zagreb hastened the development of many clinical disciplines, such as immunology, transfusion, microbiology, pathology, cytogenetics, and molecular genetics. On the other hand, without these clinical disciplines, transplantation could not be performed. The high professional level of these clinical disciplines now stands at the disposal of all other disciplines of clinical medicine. Such team approach organization and mutual interaction have enabled a number of important clinical studies. Some of them are mentioned here.

Our top scientific priority was GVHD, the most serious complication of allogeneic HSCT. One of the first clinical studies was a prospective randomized trial comparing the GVHD prophylaxis with a combination of cyclosporine (CSP) and methotrexate (MTX) to that with CSP alone. The incidence of moderate-to-severe acute GVHD was significantly higher in the CSP group compared with the CSP plus MTX group (51% vs 21%, *P* < 0.02) (72).

A prerequisite for rational treatment of GVHD is an exact and prompt diagnosis. At the beginning, the diagnosis was based on histopathologic skin classification of acute and chronic GVHD. Our preliminary data showed that an early occurrence of acute GVHD was related to a lower grade, while a late occurrence was associated with a higher grade of acute GVHD (73).

We also published a very interesting report on the influence of anxiety on the severity of acute GVHD (74). Patients who later developed acute grade II-IV GVHD had a significantly higher level of anxiety at the beginning of treatment than patients who later developed GvHD grade 0-I. The mechanisms by which anxiety acts on GVHD still remain to be elucidated.

Pavletic et al recognized the importance of GVHD for the HSCT outcome. They proposed the diagnostic criteria for GVHD, especially chronic GVHD (75-77). The exact clinical criteria help the physicians to investigate the relevance of treatment approach and compare the clinical data and outcome of chronic GVHD. In 2013, the Zagreb Center created a team for chronic GVHD, similar to the well-known team at the National Cancer Institute in Bethesda, USA.

Our team has published many relevant papers. We reported clinical laboratory markers of inflammation as predictors and determinants of chronic GvHD (78). We also investigated the relevance of novel biomarkers such as glycans, epigenetic changes (microRNAs and DNA methylation), and the microbiome (79-81). The clinical observation about the increased incidence of skin manifestations, including vitiligo and alopecia areata, is also a contribution to the clinical settings of cutaneous chronic GVHD (82). Another report showed that the application of autologous blood as a source of platelet gel in oral chronic GVHD was an efficient and safe treatment option for patients who did not respond to standard local treatment (83).

The Zagreb Transplant Team has recently participated in the creation of the Chronic GVHD Dictionary, which has been a step forward in the management of this serious complication of allogeneic HSCT. Practically all worldwide databases joined the Dictionary. The aim of this initiative is to facilitate information sharing about patient characteristics, transplant characteristics, chronic GVHD characteristics, patient-reported quality of life, symptom burden, and functional indicators (84).

We also investigated TBI conditioning. Patients with acute and chronic leukemia were randomized to receive cyclophosphamide and fractionated TBI either with or without lung shielding (85). Both groups had an identical three-year LFS (54 ± 18% without shielding vs 51% with shielding, *P* = ns). There was a trend toward a higher incidence of interstitial pneumonitis and lung fungal infection in the group without lung shielding. In conclusion, TBI without lung shielding was not superior in terms of leukemia control, but was associated with higher lung toxicity. In the future, all patients who undergo conditioning will receive fractionated TBI with lung shielding.

We also investigated ways to minimize the immediate adverse effects of aggressive conditioning for HSCT. In our prospective double-blind trial, prostaglandin E2 was not found to be effective in the control of mucositis (86). Furthermore, a microbiology laboratory specializing for fungal infections was established. The data on fungal infections in our allografted patients showed how important fungal colonization, previous antibiotic therapy, and duration of fever and skin rash were for the development of invasive fungal infections. This report also highlighted the importance of early diagnosis. A higher proportion of fungal infections were diagnosed on autopsy than during the patient's life (87).

The chimeric status might be a strong predictor of relapse in leukemia. We introduced a novel molecular technique, PCR analysis of five short tandem repeats, and found it to be highly informative for the detection of chimerism. This fast and simple method soon entered our routine clinical practice (88,89).

The Zagreb Center analyzed the effect of HLA allele and haplotype polymorphisms on donor matching for match-unrelated transplantation (90). The published study proposed the HLA factors that cause difficulties in searching for 10/10-matched unrelated donors for Croatian patients (90).

## EBMT Registry data

The EBMT Registry publishes relevant information about HSCT. This information does not only include treatment outcomes, but also valuable information about the transplant activity in different countries. The EBMT regularly publishes activity surveys for autologous, allogeneic, and unrelated HSCT in European countries. One of the surveys compared HSCT in Eastern and Western Europe from 1990 to 2003 (91). The transplant rate in Western countries was higher and reached a plateau, while no plateau was observed in Eastern countries. The plateau might be explained by the decreasing number of HSCTs for solid tumors in the autologous setting. In allogeneic HSCT, the reason for the plateau might be the decreasing number of CMLs. Countries with higher gross national income (GNI) *per capita* had higher transplant rates, but there was a saturation effect at about US$ 20 000 *per capita.* Transplant rates were also substantially lower in countries with lower team density.

The number of HSCT continues to rise (92). There is also a marked rise in CAR-T cellular therapies. In 2019, 700 centers reported to the registry, compared with 143 centers in 1990. In the 30 years, more than 800 000 HSCT in 715 000 patients were reported. Recent developments include the success of unrelated-donor and haploidentical HSCT, as well as a decrease in the number of cord blood transplants. The use of reduced-intensity HSCT in older patients has also significantly increased.

Based on the registry data, we reported the experience of HSCT for acute leukemia in several countries in Central and Eastern Europe. The probability of LFS at 60 months was 41% for AML and 39% for ALL patients. The relapse rate was higher for patients allografted at an advanced stage of the disease (47% vs 26% for AML and 55% vs 38% for ALL). Transplant-related mortality for both AML and ALL was 25%. The data confirmed that HSCT was very effective for acute leukemia treatment, especially for patients transplanted in the first CR in Central and Eastern European countries (93).

## The future of HSCT

For many years, the therapy for acute leukemia, especially for high-risk patients, was based on standard chemotherapy in combination with HSCT. To my knowledge, allogeneic HSCT with a preparative cytotoxic regimen and antitumor immune response from the donor cells is the only potentially curative treatment for high-risk acute leukemia. Despite the advances, allogeneic HSCT is still associated with significant morbidity and mortality, and many patients develop relapse. All this calls for the introduction of a novel therapy.

Recent advantages in understanding the molecular pathogenesis of acute leukemia have paved the way for novel, more precise, and less toxic therapies. The era of personalized medicine or, better to say, precision medicine starts with genomic sequencing and the development of novel molecular, immunological, and cellular therapies. The fundamental goal of precision medicine is to integrate population-based molecular, clinical, and other data to make individual-based clinical decisions for patients (94).

Clinical research into the efficacy of targeted drugs is expanding exponentially. Most of them are of limited efficacy. Although at the moment, they are not able to cure leukemia, targeted therapies have provided an important adjunct to other standard forms of treatment and HSCT. By incorporating targeted agents upfront in the treatment, one may expect higher rates of remission and deeper remission. As maintenance therapy, these agents reduce leukemia recurrence. Blinatumomab (95) and CAR T cells (96) might be potentially curative. Although the preliminary data suggest that the antileukemia immune response of allogeneic HSCT may also be achieved without transplantation, it is too early to draw definitive conclusions. We need more data if we want to establish whether the response is durable in the absence of allogeneic HSCT. Meanwhile, allogeneic HSCT remains an important therapy for patients with acute leukemia.
